# Dose escalation biodistribution, positron emission tomography/computed tomography imaging and dosimetry of a highly specific radionuclide-labeled non-blocking nanobody

**DOI:** 10.1186/s13550-021-00854-y

**Published:** 2021-10-30

**Authors:** Yanling Yang, Chao Wang, Yan Wang, Yan Sun, Xing Huang, Minzhou Huang, Hui Xu, Huaying Fan, Daquan Chen, Feng Zhao

**Affiliations:** 1 Key Laboratory of Molecular Pharmacology and Drug Evaluation, Ministry of Education, Collaborative Innovation Center of Advanced Drug Delivery System and Biotech Drugs in Universities of Shandong, School of Pharmacy, Yantai University, Yantai, 264005 People’s Republic of China; 2 SmartNuclide Biopharma Co. Ltd, 218 Xinghu St., BioBAY A4-202, Suzhou Industrial Park, Suzhou, 215123 People’s Republic of China; 3 Department of Clinical Pharmacology, First Affiliated Hospital of Soochow University, 899 Pinghai Road, Gusu District, Suzhou, 215006 People’s Republic of China; 4 Zhejiang Provincial Key Laboratory of Pancreatic Disease, Department of Hepatobiliary and Pancreatic Surgery, The First Affiliated Hospital, School of Medicine, Zhejiang University, Hangzhou, 310058 People’s Republic of China

**Keywords:** Nb109, PD-L1, PET imaging, nanobody, Tracer, ^68^Ga

## Abstract

**Background:**

Immunotherapy is a valuable option for cancer treatment, and the curative effect of anti-PD-1/PD-L1 therapy correlates closely with PD-L1 expression levels. Positron emission tomography (PET) imaging of PD-L1 expression is feasible using ^68^Ga-NOTA-Nb109 nanobody. ^68^Ga-NOTA-Nb109 was generated by radionuclide (^68^Ga) labeling of Nb109 using a NOTA chelator. To facilitate clinical trials, we explored the optimal dose range of ^68^Ga-NOTA-Nb109 in BALB/c A375-hPD-L1 tumor-burdened nude mice and C57-hPD-L1 transgenic MC38-hPD-L1 tumor-burdened mice by administration of a single intravenous dose of ^68^Ga-NOTA-Nb109 and confirmed the dose in cynomolgus monkeys. The biodistribution data of cynomolgus monkey PET images were extrapolated to estimate the radiation dose for the adult male and female using OLINDA2.1 software.

**Results:**

^68^Ga-NOTA-Nb109 was stable in physiologic media and human serum. Ex vivo biodistribution studies showed rapid and specific uptake in A375-hPD-L1 or MC38-hPD-L1 tumors. The estimated ED_50_ was approximately 5.4 µg in humanized mice. The injected mass (0.3–100 µg in nude mice and approximately 1–100 µg in humanized mice) greatly influenced the general biodistribution, with a better tumor-to-background ratio acquired at lower doses of Nb109 (0.3–10 µg in nude mice and approximately 1 µg in humanized mice), indicating maximum uptake in tumors at administered mass doses below the estimated ED_50._ Therefore, a single 15-μg/kg dose was adopted for the PET/CT imaging in the cynomolgus monkey. The highest specific and persistent uptake of the tracer was detected in the spleen, except the levels in the kidney and urine bladder, which was related to metabolism and excretion. The spleen-to-muscle ratio of the tracer exceeded 10 from immediately to 4 h after administration, indicating that the dose was appropriate. The estimated effective dose was calculated to yield a radiation dose of 4.1 mSv to a patient after injecting 185 MBq of ^68^Ga-NOTA-Nb109.

**Conclusion:**

^68^Ga-NOTA-Nb109 showed specific accumulation in hPD-L1 xenografts in ex vivo biodistribution studies and monkey PET/CT imaging. The dose escalation distribution data provided a recommended dose range for further use, and the safety of the tracer was confirmed in dosimetry studies.

**Supplementary Information:**

The online version contains supplementary material available at 10.1186/s13550-021-00854-y.

## Background

The Global Cancer Statistics report estimated 18.1 million new cancer cases and 9.6 million cancer deaths in 2018, with > 50% of the cancer deaths occurring in Asia [1]. A new report predicts a 60% increase in the global number of cancer cases within the next two decades, with lung cancer continuing to be the leading cause of cancer deaths [2]. Immunotherapy, especially inhibitors targeting programmed cell death protein 1 (PD-1) or its ligand (PD-L1), has become the focus of cancer research in recent years. As the outcome of PD-1: PD-L1 inhibitors is correlated with the PD-L1 expression status of the tumor, it is vital to detect PD-L1 expression before and during treatment.

In recent years, tracers for radioimmune imaging have provided a non-invasive alternative to traditional immunohistochemical (IHC) staining to monitor PD-L1 expression. Although ^18^F-fluorodeoxyglucose (^18^F-FDG) is the most commonly used radioactive tracer, its uptake is not tumor cell-specific, and it can also be taken up by activated immune cells [3]. Thus, several specific imaging agents, including monoclonal antibodies (mAbs; such as ^89^Zr-avelumab [4], ^89^Zr-atezolizumab [5], ^89^Zr-nivolumab [6]), mAb fragments (such as minibodies and nanobodies [7, 8]), small proteins (such as ^18^F-BMS-986192 [6, 9]), and peptides (such as ^64^Cu-WL12 [10]), have been developed and investigated in preclinical models in addition to some early clinical studies.

Although the high specificity, affinity, and ready availability of full-length IgG antibodies provide some feasibility for imaging, the characteristics of mAb metabolism in the liver lead to the high uptake of radioactivity in the liver, and their large size (approximately 150 kD) limits tissue penetration, tumor retention, and clearance from the circulation. Furthermore, the long half-lives of these proteins mean that high-contrast images cannot be obtained in a short timeframe (several days are required) [11, 12]. To overcome these challenges, peptides and smaller antibody fragments (approximately 10 kD) have been developed for in vivo imaging. These agents provide superior imaging characteristics, such as rapid clearance from the circulation, higher tissue penetration, and higher signal-to-background ratios [8].

The protein dose significantly impacts imaging. The uptake of ^68^Ga-DOTA-TOC (the first FDA-approved ^68^Ga-radiopharmaceutical for PET imaging of somatostatin receptor (SSTR)-positive gastroenteropancreatic neuroendocrine tumors) improved in the neuroendocrine tumor and decreased in the liver and spleen as the peptide dose increased to 50 µg. However, the uptake decreased in the lesions and healthy organs with a further elevation of the peptide dose to 500 µg [13]. The lesion-to-liver uptake ratio of ^68^Ga-ABY-025 (a radiolabelled affibody molecule for in vivo diagnosis of HER2-positive breast cancer tumors with PET) was higher with a high peptide dose (427 µg) than with a low peptide dose (78 µg) [14]. The spleen, blood, and tumor uptakes of ^111^In-DTPA-anti-PD-L1 were significantly altered (that of spleen reduced, and those of blood and tumor increased) in the presence of excess (30- or 100-fold) unlabeled anti-PD-L1 mAb (compared with the unblocked control group, *P* ≤ 0.0002 for spleen and blood and *P* ≤ 0.05 for the tumor in the blocked group) [15]. In a study of the PD-L1 targeting tracer ^64^Cu-WL12, the imaging and biodistribution data showed high uptake in the tumor after pretreatment with low doses (0.06 mg/kg) of the anti-PD-L1 mAb (atezolizumab whose binding interface on PD-L1 overlaps with WL12), compared to that at the higher doses of the mAb (0.6 and 3.6 mg/kg) [16]. These findings indicate that the signal-to-background uptake was high relative to the unlabeled dose of the protein.

Nanobodies derived from camelid heavy-chain antibodies, also known as single-domain antibodies, are small proteins (approximately 15 kDa). The conformation of the complementarity determining regions (CDR) of nanobodies often presents large convex paratopes that can access hidden epitopes less accessible to conventional antibodies in protein cavities [17]. Small molecular sizes confer nanobodies many features for imaging applications, such as rapid targeting, blood clearance, high solubility, stability, and ease of cloning [18]. Furthermore, their low immunogenicity risk profile provides more possibilities for the clinical translation of nanobodies [19].

Some studies have shown that the tumor PD-L1 protein levels can predict the response to PD-1/PD-L1 checkpoint block therapy in cancer patients [20–22], and the nanobody-based imaging can display the PD-L1 expression level in vivo in real-time. Thus, it would be able to predict the response for therapy with a therapeutic antibody, which has been evaluated in some studies [23–25].

Nb109 is a non-blocking nanobody with a high specific affinity for PD-L1 (with an equilibrium dissociation constant (K_D_) of 2.9 × 10^–9^ M) [24, 26]. Nb109 binds well to primate (including human and monkey) PD-L1 but does not cross-react with mouse PD-L1. The epitope to which Nb109 binds differs from that bound by the therapeutic PD-1 and PD-L1 antibodies. After conjugation with the chelator 1,4,7-triazacyclononane-1,4,7-triacetic acid (NOTA) and labeling with the radionuclide ^68^Ga, ^68^Ga-NOTA-Nb109 can bind with PD-L1 in vivo and accumulate specifically in locations with high PD-L1 expression, such as the A375-hPD-L1 tumor. The radioactive uptake is associated with the PD-L1 expression level in target organs. In addition to the uptake in the tumor, the kidney exhibited relatively high uptake compared with the liver and other organs (33.7% vs. 1.1% and < 1.5% ID/g, where %ID/g refers to the percentage of the injection dose per gram of tissue).

In this study, we explored the suitable dose of ^68^Ga-NOTA-Nb109 by comparing the effect of the injected mass on the biodistribution in two strains of mice (including humanized mice) and conducted positron emission tomography/computed tomography (PET/CT) imaging in non-human primates to further verify the dose of ^68^Ga-NOTA-Nb109 based on the protein mass dose range determined. The biodistribution data of cynomolgus monkeys were extrapolated to estimate the radiation dose for the adult male and female.

## Materials and methods

All the commercially obtained chemicals were of analytic grade, and all the reagents were obtained from Sinopharm (Shanghai, China) unless otherwise stated. p-SCN-Bn-NOTA was purchased from Macrocyclics (USA). ^68^Ga was obtained from a ^68^Ga/^68^Ge generator (ITG) and eluted with 0.1 M hydrochloric acid (HCl, Merck). High-performance liquid chromatography (HPLC) was performed using e2695 HPLC (Waters), and thin-layer chromatography (TLC) was performed on a Mini-scan TLC scanner (Eckert & Ziegler Radiopharma).

### Production and purification of Nb109

The anti-PD-L1 nanobody Nb109 was produced as described previously [24, 26].

### Conjugation of p-SCN-Bn-NOTA to the nanobody

Nb109 was produced as described previously [26]. Nb109 (5 mg/mL) in 0.5 M sodium carbonate buffer (pH = 9.8) was added to p-SCN-Bn-NOTA (sixfold molar excess) and incubated for 24 h at 30 °C. The conjugate was then purified, and the concentration of NOTA-Nb109 was determined at 280 nm using corrected extinction coefficients (*ε* = 34,654 LM^−1^ cm^−1^) based on the number of chelates per nanobody. The number of chelates per nanobody was determined by reverse-phase high-performance liquid chromatography (RP-HPLC) and electrospray ionization (Thermo Scientific™ Q Exactive™), with the final value of about 1.5. The bioactivity (binding capacity) and serum concentration of Nb109 were evaluated by enzyme-linked immunosorbent assay (ELISA) using a microplate reader (MD SpectraMax190).

### Synthesis of ^68^Ga-NOTA-Nb109

Briefly, using HCl as the eluent, the ^68^Ga radionuclide was eluted from a ^68^Ga/^68^Ge generator (Germany, ITG). The precursor (NOTA-Nb109) was mixed with ^68^Ga^3+^ in an acetate reaction system (pH = 4.0‒4.5) and incubated at room temperature to obtain a ^68^Ga-NOTA-Nb109 injection sample. After the reaction was completed, sodium acetate solution was added (pH = 5.5). Finally, the solution was filtered through a 0.22-μm sterile filter membrane. Finally, a sterile pyrogen-free formulation of ^68^Ga-NOTA-Nb109 was achieved in sodium acetate solution at about pH = 5.5. The radiochemical purity and the stability of ^68^Ga-NOTA-Nb109 were measured by radio-TLC or radio-HPLC.

In the dose escalation distribution study, the activity-to-volume ratio of the sample was calculated. The mass of Nb109 in the dosing solution was determined by its specific activity.

### Cell lines and animals

The A375-PD-L1 and MC38-PD-L1 cell lines were generated by lentivirus infection and kindly provided by SmartNuclide Biopharm (China). The cells were cultured, and the PD-L1 expression level was confirmed via flow cytometry and immunohistochemical staining as described previously [26].

Female BALB/c nude mice and female C57BL/6J-Cd274em (hPD-L1)/Smoc mice (C57-hPD-L1 transgenic mice for short) were purchased from Gem Pharmatech Co., Ltd and ShangHai Model Organisms, respectively. The mice were subcutaneously inoculated at the left axilla with A375-PD-L1 (5 × 10^6^ per mouse) or MC38-PD-L1 (0.5 × 10^6^ per mouse), respectively. The tumors were allowed to grow for 1–2 weeks to approximately 100 mm^3^.

Cynomolgus monkey studies were conducted in collaboration with WuXi AppTec (Shanghai, China) and Soochow University-SmartNuclide Radiopharmaceutical Collaborative Innovation Center (Suzhou, China).

### Ex vivo biodistribution in BALB/c nude mice

Female BALB/c A375-hPD-L1 tumor-burdened nude mice were injected with 0.3–1.2 MBq of ^68^Ga-NOTA-Nb109 via the tail vein. As described previously, the protein quantity was adjusted by diluting the starting labeled preparation (^68^Ga-NOTA-Nb109) with NOTA-Nb109 or normal saline to provide tracer doses containing 0.3 µg, 1 µg, 10 µg, and 100 µg (*n* = 6 in each subgroup). At 90 min after dosing, the mice were sacrificed to collect tumors and other organs, which were washed, weighed immediately, and assessed with a gamma counter. Tumor and tissue or organ uptake were calculated as %ID/g and corrected for decay.

### Ex vivo biodistribution and micro-PET imaging in humanized mice

To further explore the impact of injected tracer mass on specific uptake in normal and tumor tissues in the humanized system, the biodistribution of ^68^Ga-NOTA-Nb109 containing four different quantities of protein was evaluated in MC38-hPD-L1 xenografts in C57-hPD-L1 transgenic mice 90 min after injection.

Female C57-hPD-L1 transgenic tumor-burdened mice were intravenously injected with 3–3.5 MBq of ^68^Ga-NOTA-Nb109 in conjunction with escalating doses of unlabeled NOTA-Nb109 (1 µg, 10 µg, 50 µg, and 100 µg per animal, *n* = 3 in each subgroup). At 90 min after dosing, the mice were sacrificed, and tumors and tissue and organ uptake (%ID/g) were calculated and corrected for decay.

In addition, blood samples were collected from each animal 90 min after dosing and analyzed for Nb109 concentrations (ng/mL) using a microplate reader (SpectraMax Plus190, Molecular Devices).

An additional tumor-bearing animal in each dose group was anesthetized with isoflurane and injected with 4.0–5.5 MBq tracers for dynamic whole-body PET scans (approximately 3 h) using a micro-PET scanner (Siemens Medical Solutions, Germany). Seventeen frame dynamic emission images were collected at 10-min intervals. The uptake of tracers in the tumor or muscle (%ID/g) was estimated by sketching the region of interest (ROI) on images using ASIPro VM™ software (Siemens Medical Solutions, USA).

### Cynomolgus monkey PET/CT imaging

A male and a female healthy cynomolgus monkey were anesthetized by intramuscular injection of a 0.3-mL/kg dose of ketamine and transferred to the PET/CT scanner (PSTE16, GE Healthcare). After administration of ^68^Ga-NOTA-Nb109 (approximately 30 MBq, 15 μg/kg) via the posterior saphenous vein, whole-body PET/CT scanning was performed on the cynomolgus monkey under anesthesia with 0.75 mg/kg of xylarizonaine. After a low-dose CT scan (voltage: 120 kV, current: 100 mAs, 3.75 mm slice thickness, 512 × 512 matrix, and 50-cm DFOV), emission images were acquired in a sequence of five passes over the previous five bed positions (5 min per position), producing a series of whole-body images covering approximately 240 min after tracer administration.

After the scan, the images (immediately, 15 min, 30 min, 60 min, 90 min, 120 min, and 240 min post-dose) were reconstructed iteratively. The reconstructed images were processed using medical analysis software PMOD to outline the ROI in tissues such as the heart, lungs, liver, kidneys, spleen, brain, muscles, and bladder. The SUV (standard uptake value) in the ROI region was calculated.

Furthermore, blood samples were collected pre-dose and 5 min, 15 min, 30 min, 60 min, 90 min, 120 min, 180 min and 240 min post-dose to determine blood radioactivity using a gamma counter (PE 2480). The remaining blood samples were then centrifuged at 4 ºC to obtain serum for Nb109 concentration (ng/mL) analyses by ELISA. The absorbance in each well was measured at 450/650 nm using a microplate reader (SpectraMax Plus190, Molecular Devices). Intact ^68^Ga-NOTA-Nb109 signals in serum samples (approximately 100 μL) isolated from blood samples and collected at 15 min, 30 min, and 60 min post-dose were subjected to radio-HPLC analyses.

### Statistical analyses

The NCA model was employed to analyze the PK parameters of plasma concentration, blood %ID/g, and heart ROI-SUV (Phoenix WinNonlin, version 8.1). A two-sided test was used to ascertain the significant differences in the distribution of data between groups.

## Results

### Synthesis of ^68^Ga-NOTA-Nb109

The radiochemical purity of ^68^Ga-NOTA-Nb109 measured by radio-TLC exceeded 95%. The in vitro stability of ^68^Ga-NOTA-Nb109 in human serum and 0.9% NaCl solution was demonstrated by radiochemical purity of > 95% over 240 min at < 25 °C. The urine analysis of ^68^Ga-NOTA-Nb109 in ICR mice after a single intravenous injection showed the high in vivo stability by the radiochemical purity results of > 93% within 120 min and 82.27% at 240 min, indicating the excretion mainly in the parental form (Additional file 1: Fig. S1).

### Ex vivo distribution in rodents

#### Distribution in BALB/c nude mice

After a single intravenous dose of ^68^Ga-NOTA-Nb109 in BALB/c-A375-hPD-L1 model mice, the analysis of the uptake in each group showed specific binding in A375-hPD-L1 tumors, with the greatest uptake of the tracer (%ID/g) in the kidney.

**Fig. 1:**
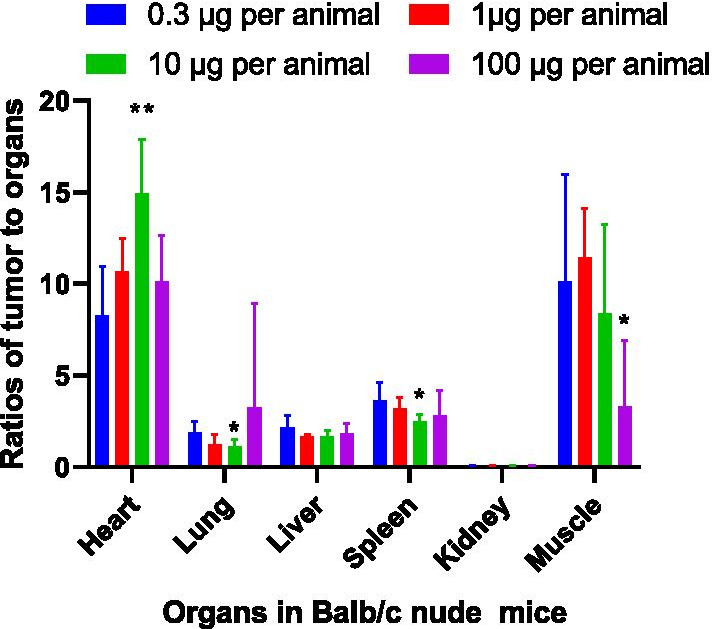
Tumor-to-organ ratios for different masses of ^68^Ga-NOTA-Nb109 in Balb/c A375-hPD-L1 tumor-bearing nude mice (*n* = 6) at 90 min after the tracers injection. Data represent the mean ± SD (**P < 0.01, *P < 0.05). *P* values were calculated vs. the 0.3-μg group by a two-sided test

The A375-hPD-L1 tumor-to-muscle ratio in the 0.3–10 μg/animal dose group was approximately 2.5–3.5 times greater than that in the 100 μg/animal dose group. In addition, the tumor-to-organ ratio changed (heart increased; lungs, liver, spleen, and kidneys decreased) in a dose-dependent manner in the 0.3–10 μg/animal group, while a decrease in the uptake in tumors was observed in the 100 μg/animal group (Fig. 1). Compared with the 0.3 μg/animal group, the ratio of tumor-to-muscle in the 100 μg/animal group decreased significantly, indicating that PD-L1 binding was inhibited by an excess of unlabeled NOTA-Nb109.

#### Distribution in C57-hPD-L1 transgenic mice

**Fig. 2:**
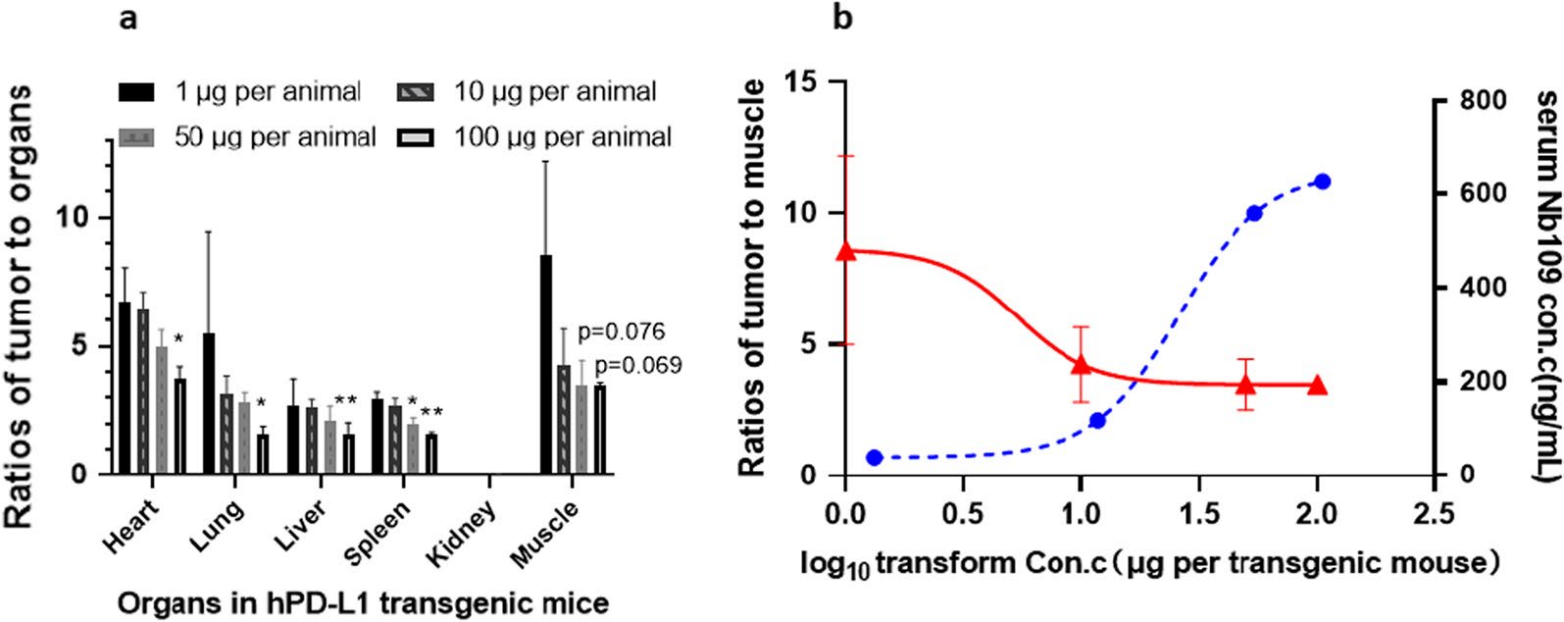
**a** Tumor-to-organ ratios for different masses of ^68^Ga-NOTA-Nb109 in C57-hPD-L1 transgenic MC38-hPD-L1 tumor-bearing mice (*n* = 3) at 90 min after the tracers injection. **b** Fitting curves of tumor-to-muscle ratios or serum Nb109 concentration (**P < 0.01, *P < 0.05). *P *values were calculated vs. 1 μg group by a two-sided test

The tumor-to-organ (heart, lungs, spleen, and liver) radioactivity ratios decreased progressively with increasing amounts of unlabeled NOTA-Nb109 (1–100 µg). The tumor-to-muscle ratio in the 1-µg group was about 2.5-fold of that in the 100-µg group, the tumor-to-lungs ratio was about 3.5-fold, and the tumor-to-other organs ratio was approximately twofold of that in the 100-µg group (Fig. 2a), indicating specific binding of the tracer to PD-L1 in humanized mice. Based on the tumor-to-muscle ratio of the four groups, the estimated half-maximal effective dose (ED_50_) was 5.4 µg/animal (Fig. 2b). Maximum uptake in tumors was found at an administered mass peptide dose of 1 µg/animal, which was below the estimated ED_50_. Only minor levels of tracer uptake (30–40%) remained when the PD-L1 epitope was inhibited by the administration of NOTA-Nb109 at 100 µg/animal, further indicating the inhibitory effect of unlabeled NOTA-Nb109. The raw biodistribution data of ^68^Ga-NOTA-Nb109 in C57-hPD-L1 tumor-bearing transgenic mice were presented in Additional file 1: Fig. S2.

**Fig. 3:**
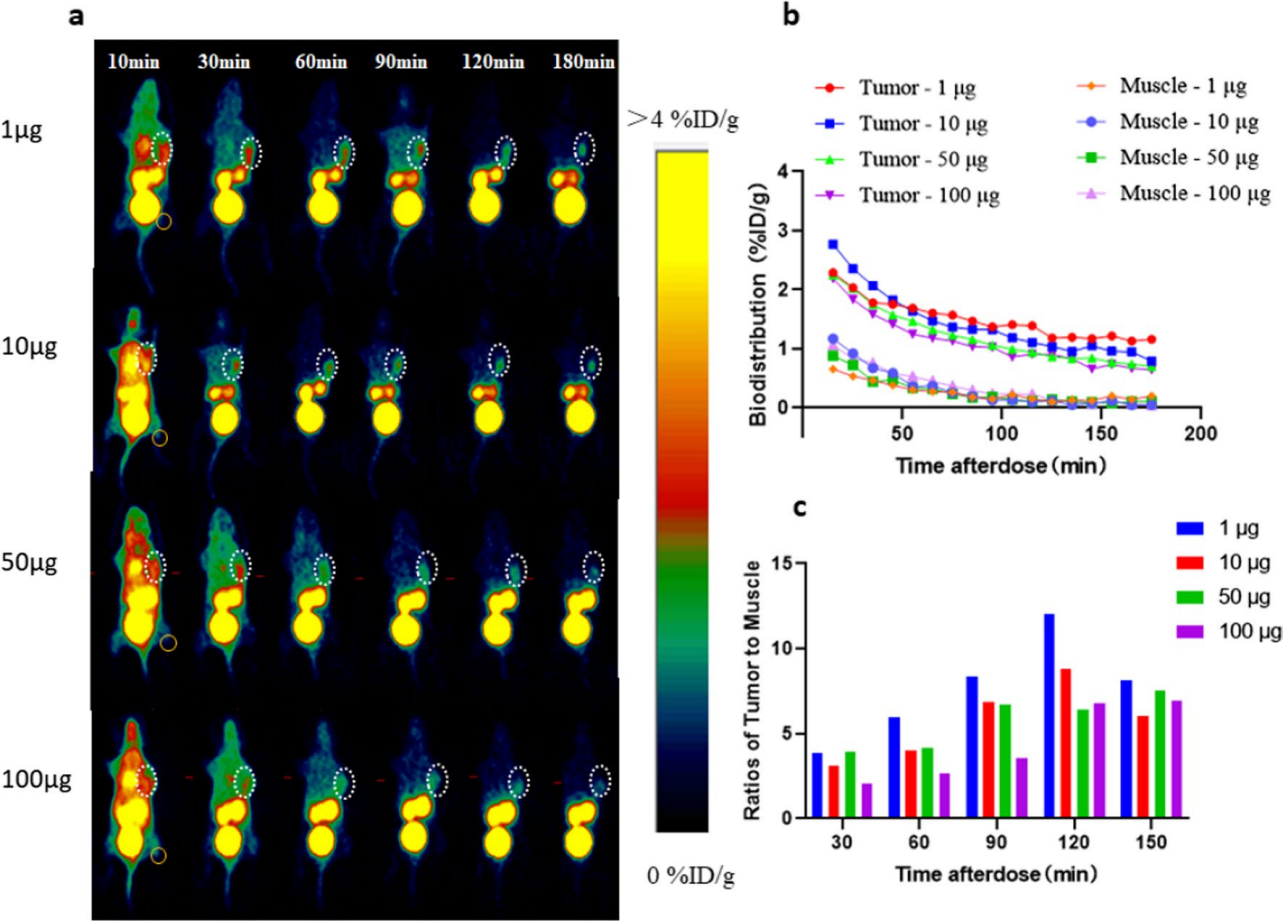
**a** Dynamic micro-PET imaging of ^68^Ga-NOTA-Nb109 in C57-hPD-L1 tumor-bearing transgenic mice over 0–3 h. **b** Biodistribution data of ^68^Ga-NOTA-Nb109 in the tumor and muscles were analyzed by quantification analysis of PET image. **c** The Tumor-to-muscle (T/M) ratio of ^68^Ga-NOTA-Nb109 was analyzed by quantification analysis of PET image. The white dotted circle indicates the tumor. The orange circle indicates the muscle

PET imaging of MC38-hPD-L1 xenografts showed a notably higher accumulation of ^68^Ga-NOTA-Nb109 at the tumor site. A good delineation of the tumor persisted until the end of the imaging session after administration at the dose of 1 µg/animal (Fig. 3a). The tumor uptake in different protein dose groups was dose-dependent from about 0.5 h post-dose to the end of scanning and was higher than the muscle uptake (Fig. 3b). The highest tumor-to-muscle ratio in the 1 µg dose group was approximately 12 (Fig. 3c).

### In vivo distribution in non-human primates

#### PET imaging

**Fig. 4:**
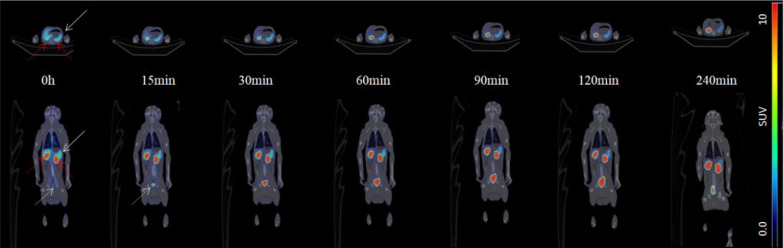
Representative PET/CT images of a healthy cynomolgus monkey at different time intervals after injecting ^68^Ga-NOTA-Nb109. Below: coronal tomograms; above: transverse tomograms. The white arrow indicates the spleen, the white dotted line indicates the bladder, and the red dotted line indicates the kidneys

**Fig. 5:**
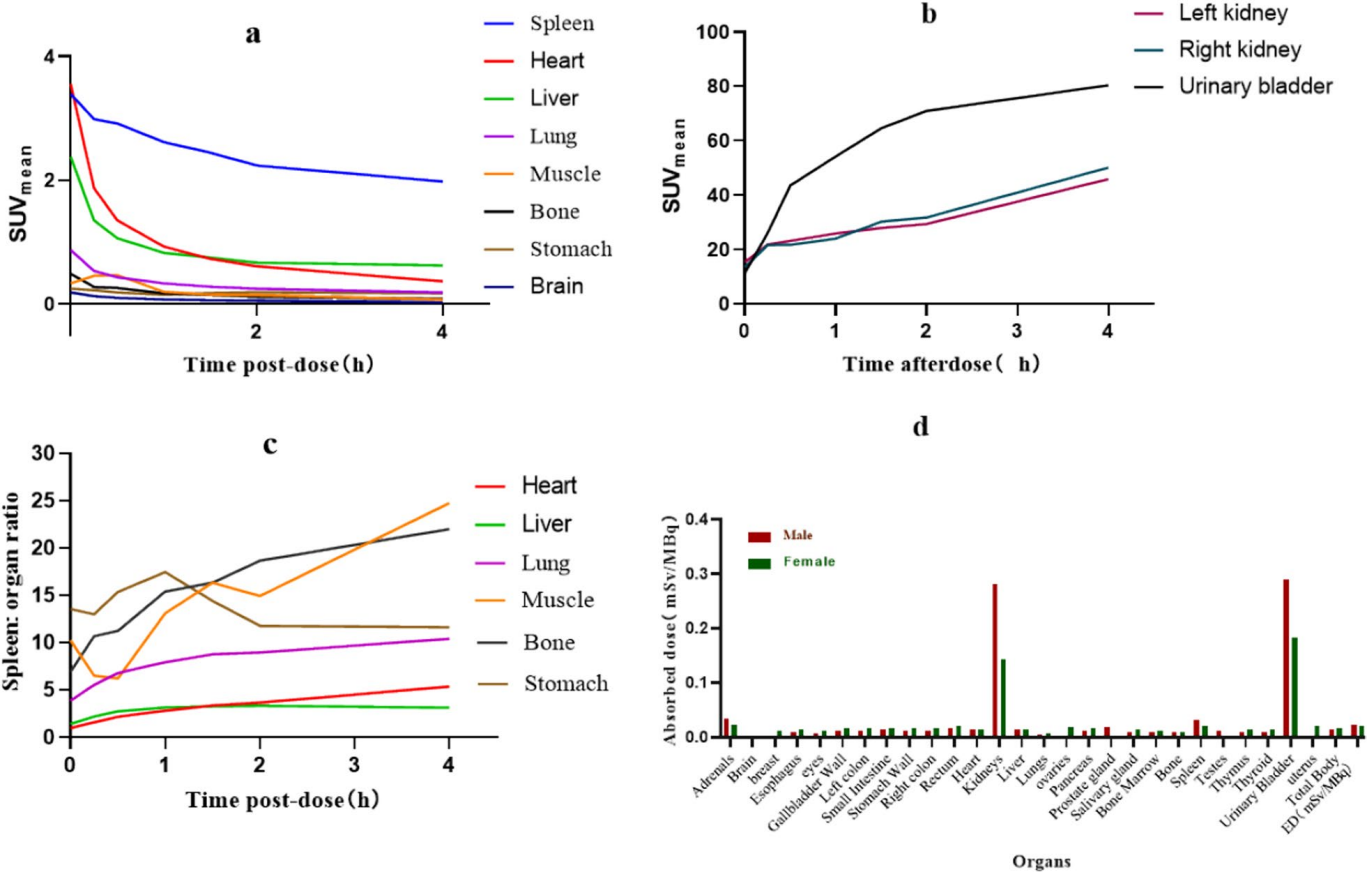
Radioactivity in the ROI in images of the male cynomolgus monkeys was quantified as SUV mean. **a** Time-activity curves of the spleen, heart, liver, lungs, muscles, bones, stomach, and brain. **b** Time-activity curves of the kidneys and urinary bladder. **c** Spleen-to-organ (heart, liver, lungs, muscles, bones, and stomach) ratio curves at different time intervals. **d** Effective dose estimation from cynomolgus monkeys to adult humans (mSv/MBq); data of each organ shows the total effective dose

As shown in Figs. 4 and 5a, b, PET scans of male cynomolgus monkeys after a single intravenous injection of ^68^Ga-NOTA-Nb109 showed that the SUV value of the spleen decreased slowly from immediately (0 h) to 240 min post-administration. Radioactive uptake was low in the brain, bones, muscles, stomach, and lungs. The uptake was high in the heart and liver immediately after dosing and then decreased rapidly to approximately 0.9 at 1 h. The SUVs of both kidneys and bladder increased rapidly over time (from about 11–15 to 45–80 during the 4-h test period after administration), indicating metabolism in the kidneys and excretion in urine. As shown in Fig. 5c, the spleen-to-muscle SUV ratio was approximately 10–12:1 from immediately to 2 h after administration, and this ratio increased to approximately 20.6 due to the decrease in the background SUV in muscles 4 h after administration. Radioactivity in the ROI in the images of female cynomolgus monkeys (quantified as SUV mean) is presented in Additional file 1: Fig. S3a–c.

#### Dosimetry studies

Organ-absorbed doses were estimated according to the ^68^Ga-NOTA-Nb109 biodistribution data of cynomolgus monkeys at different time intervals using OLINDA2.1 software and extrapolated to estimate the adult male and female equivalent. The organ-absorbed doses are summarized in Fig. 5d. The effective dose was estimated at 0.0226 mSv/MBq for male monkeys and 0.0223 mSv/MBq for female monkeys; therefore, a proposed patient dose of 185 MBq of ^68^Ga-NOTA-Nb109 would yield a radiation dose of 4.1 mSv.

#### Pharmacokinetics

The correlation coefficients between the serum concentration (ng/mL) analyzed by ELISA and ex vivo blood radioactivity (%ID/g) or heart uptake (in vivo) were 0.985 and 0.997, respectively, indicating strong correlations between the serum concentration, blood, and heart uptake (%ID/g) (Additional file 1: Fig. S4a–f). Hence, the in vivo pharmacokinetics (PK) of drug metabolism can be monitored by both in vitro methods (serum radioactivity %ID/g detection or blood Nb109 protein concentration) and in vivo methods (heart SUV) (Pharmacokinetics parameters are presented in Additional file 1: Table S1).

Serum radioactive signals (intact ^68^Ga-NOTA-Nb109) were detected by radio-HPLC at 15 min and 30 min post-dose, but the radioactivity was low at 60 min post-administration and there was barely any radioactive signal, indicating that the rapid metabolism and ^68^Ga-NOTA-Nb109 were mostly present in the parental form in the blood (Additional file 1: Fig. S5).

## Discussion

Immunotherapy is a valuable treatment strategy for cancers, especially the immune checkpoint PD-1/PD-L1-based immunotherapy [27, 28]. However, the curative effect of anti-PD-1/PD-L1 therapy is closely related to PD–L1 expression level [10]; therefore, the desired response is not achieved in all patients [27, 29]. Consequently, monitoring of tumor-based PD-L1 biomarkers has provided an important reference for therapeutic selection and prediction of the response to targeted therapies.

Previous studies [24, 26] have confirmed that the tracer ^68^Ga-NOTA-Nb109 is suitable for the specific targeting of endogenous PD-L1 and real-time detection and quantification of PD-L1 expression in different cancers. To further clarify the influence of the protein mass on this tracer, we designed a series of studies to explore the biodistribution of ^68^Ga-NOTA-Nb109 at different masses in tumor-bearing nude mice and hPD-L1 humanized mice and confirmed the selected dose in non-human primates.

The injected protein mass (Nb109) influences the uptake of ^68^Ga-NOTA-Nb109 in normal organs and tumor tissues. In nude mice, the A375-hPD-L1 tumor-to-muscle ratio in the 0.3–10 μg/animal dose group was approximately 2.5–3.5 times greater than that in the 100 μg/animal group, indicating that ^68^Ga-NOTA-Nb109 has a better target-to-background ratio at low doses. As there is no cross-reactivity between murine PD-L1 and Nb109 (data not shown), only the binding to A375-hPD-L1 tumors was regarded as specific. The non-specific uptake in other organs was influenced by the protein mass, and a dose-dependent target-to-background uptake ratio was identified. The strategy of adding unlabeled proteins to tracers to change the non-specific binding in non-target organs was also adopted for ^68^Ga-NOTA-2Rs15d in a previous study, which showed increasing specific uptake of the radioactive probe in the tumor and decreased non-specific uptake in normal organs (lungs, spleen, liver), with increasing mass of the “cold” protein from 0.1 to 10 mg [30].

Due to the high non-tumor (i.e., lymphoid tissue) expression of PD-L1, the effect of the specific binding of other normal organs on the target-to-background ratio should not be underestimated. Therefore, we further explored the influence of differences in protein mass on distribution in hPD-L1 transgenic mice using a similar dose with that mentioned above. The tumor-to-normal organ ratios decreased as the administered cold mass increased. Maximum uptake in tumors occurred after the administration of 1 µg/per animal, which was similar to the dose range observed in nude mice. These observations indicated that tumor uptake improved at doses below the estimated ED_50_. A dose escalation (0–500 μg) study using another PD-L1 tracer, ^89^Zr-DFO-6E11, co-injected with 6E11, increased the relative tumor uptake and decreased the splenic uptake [31]. One point needs to be made here. hPD-L1 humanized mice were obtained by inserting the coding gene sequence of human PD-L1 protein into the murine PD-L1 gene position to replace the expression of murine endogenous PD-L1 while expressing the whole human PD-L1 protein under the genetic background of C57BL/6J. Compared with nude mice, hPD-L1 humanized mice would be a better animal choice for the distribution studies of hPD-L1 antibodies. Theoretically, the distribution data of hPD-L1 nanobody Nb109 (binds well to primate PD-L1 but does not cross-react with murine PD-L1) in C57-hPD-L1 transgenic mice should be similar to that of murine PD-L1 nanobody (binds well to murine PD-L1) in C57 mice. Compared with the ^99m^Tc-Nbs C3–C7 [7], the radioactive uptake of ^68^Ga-NOTA-Nb109 in the liver, lung, and heart was similar to ^99m^Tc-Nbs. However, the spleen radioactive uptake did not show a similar trend, which might be attributed to the poor hPD-L1 gene transfection efficiency, leading to the subsequent low hPD-L1 expression level in transgenic hPD-L1 mice [32, 33]. Thus, since hPD-L1 is the only humanized gene in the above transgenic mice, non-human primates will be employed in further studies (such as in vivo pharmacodynamics and future toxicity studies), considering the genetic similarity between human and non-human primates and strong binding of monkey PD-L1 to Nb109.

Based on the protein mass dose of 1 µg/animal in humanized mice and an estimated body weight of 20 g for each mouse, the mouse-monkey dose conversion factor (mg/kg) was 0.25; therefore, the recommended dose for cynomolgus monkey was 12.5 µg/kg. Hence, we conducted PET/CT imaging in a cynomolgus monkey to further verify the in vivo specific binding of ^68^Ga-NOTA-Nb109 at a dose of 15 µg/kg. According to some studies [34–36], the PD-L1 expression is high in the spleen and lymph nodes; therefore, a significant uptake was detected in the spleen, although the highest uptake was detected in kidneys and urinary bladder in the present study as expected. There was no noticeable uptake in other tissues 30 min post-dose, and the lymph nodes were not visualized. The specific uptake in the spleen of cynomolgus monkeys indicates that ^68^Ga-NOTA-Nb109 binds monkey PD-L1 specifically and can be used for effective measurement of PD-L1 expression in primates in vivo*.* The high spleen-to-muscle ratio further confirmed the specificity, indicating that ^68^Ga-NOTA-Nb109 data obtained in non-human primates will provide reliable information for predicting future applications in humans. The failure to visualize lymph nodes was similar to [^18^F]BMS-986192 (anti-PD-L1 adnectin), which has been successfully used for same-day PD-L1 PET clinical imaging [6]. The high uptake in the kidneys and bladder is attributed to the metabolism and excretion of ^68^Ga-NOTA-Nb109, consistent with previous reports that attribute the high nanobody signals in the kidney to their clearance from the blood via the urine [7]. The characteristics of urine production might be a commonality of the low molecular weight protein or other molecules, such as WL12 (a ^64^Cu-labeled peptide that binds, with low nM affinity, to human, but not to murine PD-L1), NOTAZPD-L1_1 (affibody molecule with affinities of 1 nM for human and rhesus PD-L1) [37] and the ^18^F-labeled anti-PD-L1 adnectin (^18^F-BMS-986192, approximately 10 kD) [35].

Non-human primates are the ideal model of the human system; therefore, the absorbed dose was estimated based on the biodistribution data of the cynomolgus monkey. The dose-limiting organ in adult male human patients is the urinary bladder wall (0.29 mSv/MBq). The estimated absorbed dose converted from the male and female cynomolgus monkey to the adult male was about 0.0226 mSv/MBq (effective dose), and 0.0223 mSv/MBq for that of the female. The recommended dose of ^68^Ga-NOTA-Nb109 for clinical trials is approximately 185 MBq (5 mCi), which generates an effective dose of approximately 4.1 mSv. This absorbed dose is similar to the effective dose of ^68^Ga-NOTA-2Rs15d [25] (4.6 mSv for 107 MBq of administered activity) but lower than the effective dose of standard ^18^F-FDG measured by PET scanning [38] (7 mSv for 370 MBq of administered activity) and much lower than that of a ^89^Zr-labeled antibody [39] measured by PET scanning (40 mSv for 74 MBq). Thus, these data indicate that ^68^Ga-NOTA-Nb109 can be regarded as safe for clinical diagnostic translation.

In previous studies, the high stability of ^68^Ga-NOTA-Nb109 was confirmed by its integrity in physiologic media and human serum (Additional file 1: Fig. S1). Furthermore, we have proved in other studies (data not published) that the binding affinity to hPD-L1 and the metabolic characteristics of Nb109 in vivo are affected by neither the NOTA chelator conjugation nor Ga complexation (data not shown). The good consistency among heart SUV, serum radioactivity (%ID/g), and serum Nb109 protein concentration demonstrated that the in vivo PK of drug metabolism could be monitored by both in vitro methods (serum radioactivity %ID/g detection or blood Nb109 protein concentration monitoring) and in vivo methods (SUV of the heart). This would be important for clinical translation in the future.

Although the relationship between PD-L1 level and the radioactive uptake has not been thoroughly investigated, previous studies [24, 26] have indicated that ^68^Ga-NOTA-Nb109 accumulation accurately reflects the dynamic changes of PD-L1 in real-time. However, this remains to be confirmed in future studies.

## Conclusion

^68^Ga-NOTA-Nb109 showed specific accumulation in xenografts in ex vivo biodistribution studies and monkey PET/CT imaging. Based on the dose escalation distribution data, we recommended a dose range for further clinical use, and monkey dosimetry studies confirmed the safety of the tracer**.** Further quantitative studies might be required for the clinical translation of ^68^Ga-NOTA-Nb109.

## Supplementary Information

**Additional file 1.** Additional data on stability, pharmacokinetics and biodistribution analysis of transgetic mice or cynomolgus monkeys.

## Data Availability

All data generated or analyzed during this study are included in this published article [and its Additional file 1].

## Abbreviations

PET/CT: Positron emission tomography/computed tomography
PD-1: Programmed cell death protein 1
IHC: Immunohistochemical/immunohistochemistry
mAbs: Monoclonal antibodies
KD: Equilibrium dissociation constant
NOTA: 1,4,7-Triazacyclononane-1,4,7-triacetic acid
HPLC: High-performance liquid chromatography
TLC: Thin-layer chromatography
ELISA: Enzyme-linked immunosorbent assay
%ID/g: Percentage of injection dose per gram of tissue
ROI: Region of interest
SUV: Standard uptake value
ED_50_: Estimated half-maximal effective dose
T/M: Tumor-to-muscle
PK: Pharmacokinetics
