# Supplementary material for: Dose escalation biodistribution, positron emission tomography/computed tomography imaging and dosimetry of a highly specific radionuclide-labeled non-blocking nanobody
Source: EJNMMI Res. 2021 Oct 30;11:113. doi: 10.1186/s13550-021-00854-y (PMC8557220; doi:10.1186/s13550-021-00854-y)
Supplement: Supplementary file 1 — Additional file 1. Additional data on stability, pharmacokinetics and biodistribution analysis of transgetic mice or cynomolgus monkeys. [file 13550_2021_854_MOESM1_ESM.docx]

**Additional file 1: Dose escalation biodistribution, positron emission tomography/computed tomography imaging and dosimetry of a highly specific radionuclide-labeled non-blocking nanobody**

**Authors**

Yanling Yang^1^, Chao Wang^2^, Yan Wang^3^, Yan Sun^2^, Xing Huang^4^, Minzhou Huang^3^, Hui Xu^1^, Huaying Fan^1^, Daquan Chen^1^, Feng Zhao^1^

**
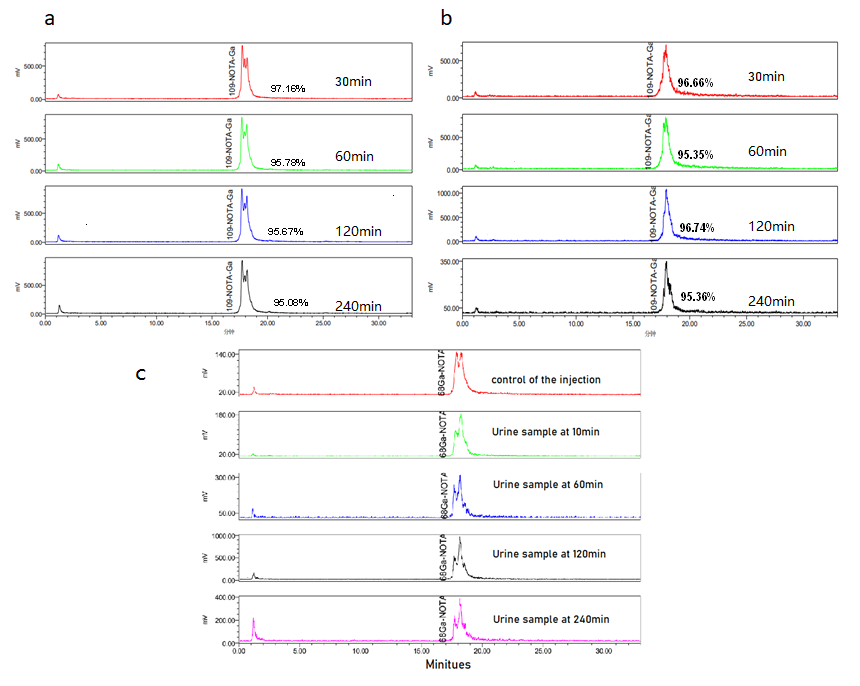
Supplemental Figure 1** *In vitro* and *in vivo* stability determined by radio-HPLC of ^68^Ga-NOTA-Nb109. (a) In human serum (*in vitro*); (b) In 0.9% NaCl solution; (c) In urine of ICR mice (*in vivo*).

**Supplemental Figure 2** Biodistribution analysis at 1.5 h post-injection of ^68^Ga-NOTA-Nb109 in C57-hPD-L1 tumor-bearing transgenic mice.


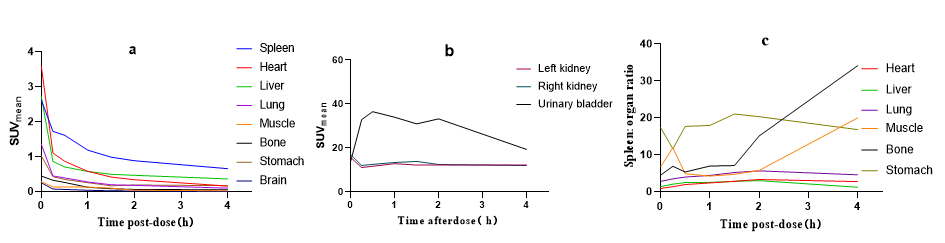
**Supplemental Figure 3** Radioactivity in the ROI in images of female cynomolgus monkeys was quantified as SUV mean. (a) Time-activity curves of the spleen, heart, liver, lungs, muscles, bones, stomach, and brain; (b) Time-activity curves of the kidneys and urinary bladder; (c) Spleen: organ (heart, liver, lungs, muscles, bones, and stomach) ratio curves at different time intervals.


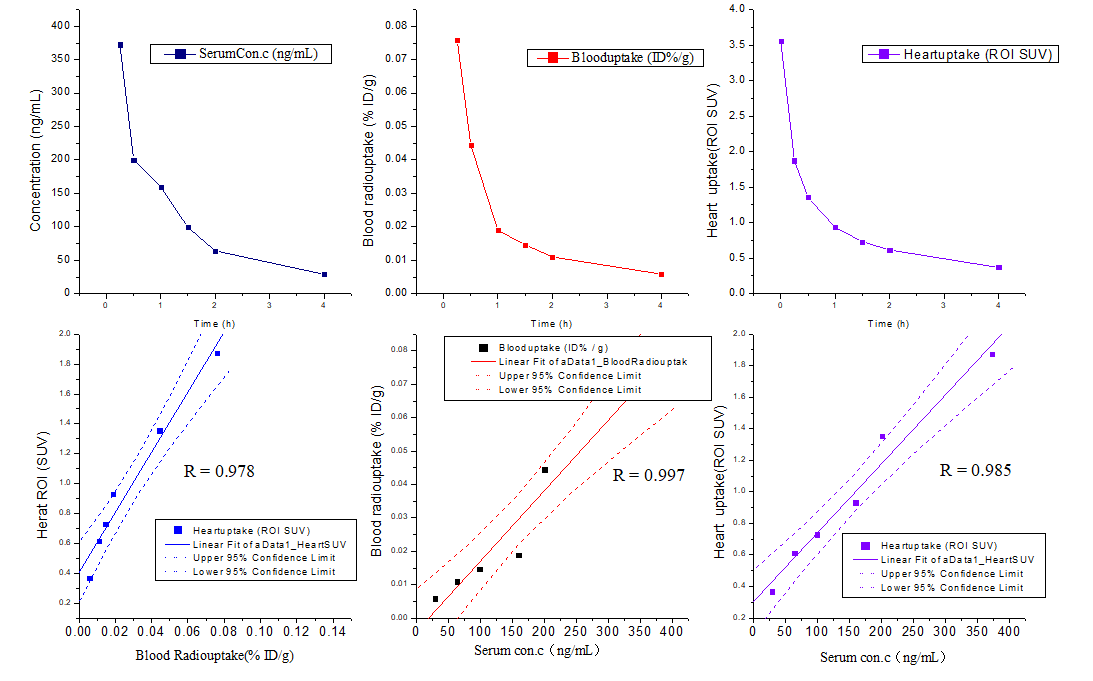


**Supplemental Figure 4.** Pharmacokinetic analysis in the cynomolgus monkey. (a-c) Curves show the serum concentration, blood, and heart uptake over time, analyzed by ELISA, gamma counter, and PET/CT imaging, respectively. (d-f) Pearson’s correlation analysis showed linear correlations among the above three indexes.


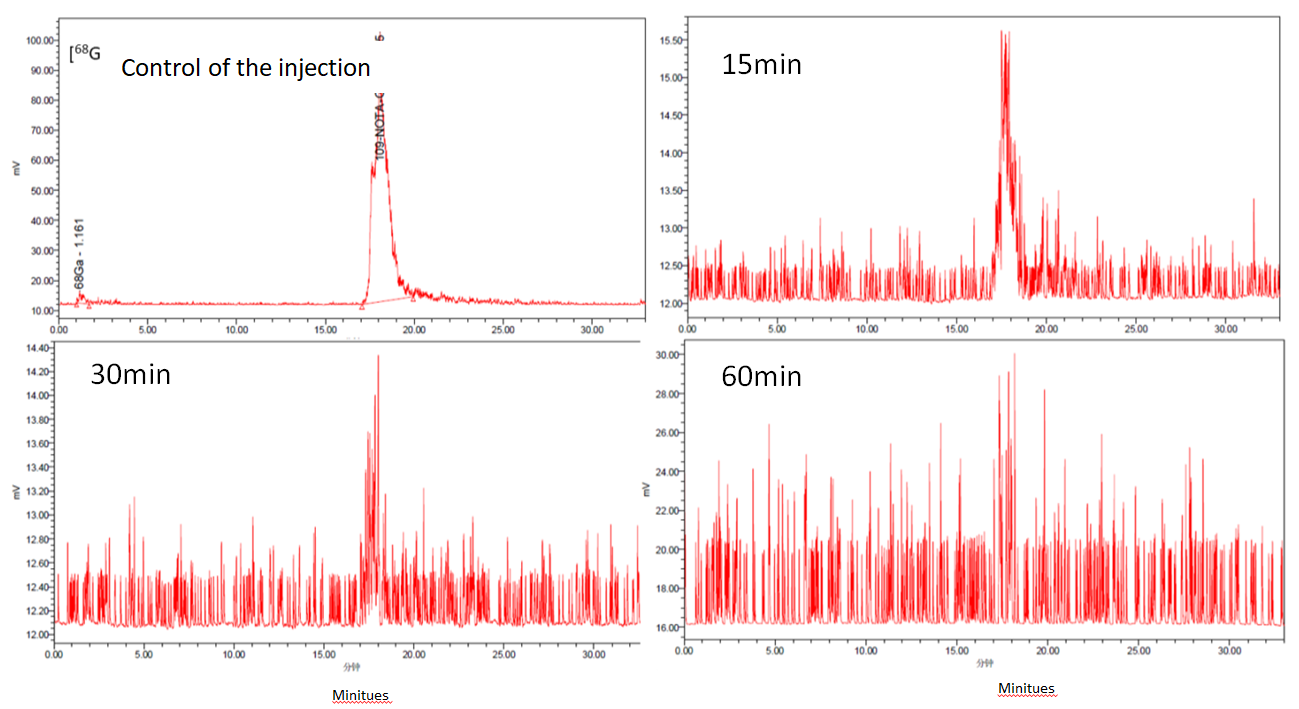


**Supplemental Figure 5** Serum radio-HPLC and radio-TLC analysis of cynomolgus monkeys after a single intravenous injection of ^68^Ga-NOTA-Nb109.

**Supplemental Table 1** Pharmacokinetic parameters for cynomolgus monkeys

| Parameters | t1/2 (h) | C_0_ | AUC_0–last_ | AUC_0–inf_ | MRT_last_ |
| --- | --- | --- | --- | --- | --- |
| Protein concentration (ng/mL) | 1.24 | 693.16 | 493.23 | 544.96 | 1.00 |
| Blood (ID%/g) | 1.81 | 0.13 | 0.09 | 0.10 | 0.95 |
| Heart (SUV) | 2.57 | 3.56 | 3.39 | 4.75 | 1.30 |

**Note:** Apparent terminal elimination half-life (t1/2); Initial concentration (C_0_), the area under the concentration–time curve (AUC) from time 0 to the time when the final concentration was measured (AUC_0–last_), and AUC was extrapolated to infinity (AUC_0–inf_), mean residence time from time 0 to the time at which the lowest serum drug concentration can be detected (MRT_last_).
